# Comorbid Prolonged Grief, PTSD, and Depression Trajectories for Bereaved Family Surrogates

**DOI:** 10.1001/jamanetworkopen.2023.42675

**Published:** 2023-11-10

**Authors:** Fur-Hsing Wen, Holly G. Prigerson, Wen-Chi Chou, Chung-Chi Huang, Tsung-Hui Hu, Ming Chu Chiang, Li-Pang Chuang, Siew Tzuh Tang

**Affiliations:** 1 Department of International Business, Soochow University, Taipei, Taiwan; 2 Department of Medicine, Weill Cornell Medicine, New York, New York; 3 Division of Hematology-Oncology, Chang Gung Memorial Hospital at Linkou, Tao-Yuan, Taiwan; 4 College of Medicine, Chang Gung University, Tao-Yuan, Taiwan; 5 Department of Internal Medicine, Division of Pulmonary and Critical Care Medicine, Chang Gung Memorial Hospital at Linkou, Tao-Yuan, Taiwan; 6 Department of Respiratory Therapy, Chang Gung University, Tao-Yuan, Taiwan; 7 Department of Internal Medicine, Division of Hepato-Gastroenterology, Chang Gung Memorial Hospital at Kaohsiung, Kaohsiung, Taiwan; 8 Department of Nursing, Chang Gung Memorial Hospital at Kaohsiung, Kaohsiung, Taiwan; 9 School of Nursing, Medical College, Chang Gung University, Tao-Yuan, Taiwan; 10 Department of Nursing, Chang Gung University of Science and Technology, Tao-Yuan, Taiwan

## Abstract

**Importance:**

Family surrogates of patients who die in an intensive care unit (ICU) are at risk of cooccurring prolonged grief disorder (PGD), posttraumatic stress disorder (PTSD), and depressive disorder during bereavement, but symptom trajectories are often explored individually.

**Objectives:**

To simultaneously examine and determine co-occurrence of PGD, PTSD, and depressive symptom trajectories.

**Design, Setting, and Participants:**

This cohort study was conducted in ICUs of 2 Taiwanese medical centers from January 2018 to March 2020, with follow-up through July 2022. Participants included surrogates responsible for decision-making who provided data 6 to 24 months after the death of their loved one. Data were analyzed from August to December 2022.

**Main Outcomes and Measures:**

PGD was assessed with the 11 grief symptom items of the PG-13; PTSD, the Impact of Event Scale–Revised; and depressive symptoms, the depression subscale of the Hospital Anxiety and Depression Scale at 6, 13, 18, and 24 months after the death. Latent growth mixture modeling was conducted to identify distinct trajectories, and joint latent class analysis was used to assess joint patterns of trajectories.

**Results:**

A total of 303 participants were included, with most younger than 56 years (207 participants [68.3%]), female (177 participants [58.4%]), and married (228 participants [75.2%]), and their relationship with the patient was mostly spouse (88 participants [29.0%]) or adult child (166 participants [54.8%]). Three trajectories were identified each for PGD, PTSD, and depressive symptoms. A resilience trajectory was predominant across PGD (253 participants [83.5%]), PTSD (250 participants [82.5%]), and depressive (200 participants [66.0%]) symptoms. Second most common was a recovery trajectory identified for PGD (36 participants [11.9%]) and PTSD (41 participants [13.5%]) symptoms, while for depressive symptoms, a moderate trajectory (72 participants [23.8%]) signified persistent moderate distress. A chronic trajectory characterized by persistently high distress was identified for PGD (14 participants [4.6%]) and depressive (31 participants [10.2%]) symptoms, whereas a unique delayed-onset trajectory (12 participants [4.0%]) was identified for PTSD symptoms. Most family surrogates (228 participants [75.2%]) experienced cooccurring PGD, PTSD, and depressive symptom trajectories, but multiple patterns were discordant. Symptom trajectories cooccurred in joint patterns: resilient (247 participants [81.5%]), recovered (43 participants [14.1%]), and distressed (14 participants [4.5%]). These patterns were characterized by high conditional probabilities for the resilience (PGD, 0.999; PTSD, 0.999; depressive, 0.804), recovery (PGD, 0.854; PTSD, 0.890; depressive, 0.588), and chronic (PGD, 0.921; PTSD, 0.789; depressive, 0.980) symptom trajectories.

**Conclusions and Relevance:**

In this cohort study, grief-related psychological symptoms evolved in complex ways during ICU bereavement, as characterized by heterogeneous trajectories. Some ICU bereaved surrogates experienced persistent elevated PGD, PTSD, and depressive symptoms individually or conjointly, underscoring the importance of early screening to identify this population at high risk of comorbid psychological distress trajectories.

## Key Points

**Question:**

How do symptom trajectories of prolonged grief disorder (PGD), posttraumatic stress disorder (PTSD), and depression cooccur among bereaved family surrogates of patients who died in intensive care?

**Findings:**

In this cohort study including 303 bereaved family surrogates, symptom trajectories cooccurred in joint patterns, with most participants falling in the resilient pattern, then recovered, then distressed. Patterns showed high conditional probabilities for the resilience, recovery (with moderate depressive symptoms), and chronic (with delayed-onset PTSD) PGD, PTSD, and depressive symptom trajectories, respectively.

**Meaning:**

These findings suggest that grief-related psychological symptoms evolved in complex ways, and some bereaved surrogates conjointly experienced persistent elevated grief symptoms, underscoring the importance of early screening to identify this high-risk population.

## Teaser

This cohort study examines the prevalence and co-occurrence of prolonged grief disorder, posttraumatic stress disorder (PTSD), and depressive symptom trajectories among family surrogates of patients who died in intensive care in Taiwan.

## Introduction

Death occurs frequently^1^ and increasingly^2^ in hospital intensive care units (ICUs), especially since the COVID-19 pandemic.^3^ Although bereavement is one of the most stressful universal human experiences,^4^ severity and duration are markedly heterogenous.^5^ Most bereaved individuals are resilient and experience transient emotional perturbations, whereas some individuals experience profound psychological distress,^5,6^ eg, prolonged grief disorder (PGD),^7^ posttraumatic stress disorder (PTSD),^8^ and depressive disorder.^9^ Two classic systematic reviews^5,10^ on outcomes of adversity identify consistent trajectories: resilience (ie, minimal impact), recovery, chronic, and delayed onset, with resilience as the modal response.^5,10^ Distinct trajectories of grief reactions (eg, PGD, PTSD, and depressive symptoms) differ in bereavement consequences, including for health, functioning, and mortality,^11,12,13^ highlighting the need to identify bereavement outcome trajectories.

Most existing studies among adults focus on symptom trajectories for PGD,^12,13,14,15,16,17^ PTSD,^18,19,20,21^ and depression^22,23,24,25,26^ individually without considering concurrent changes in emotional distress from bereavement and traumatic events. However, distressed psychological grief reactions cooccur as the norm rather than the exception,^27^ and resilience in 1 outcome (eg, PGD) may often coexist with deficits in other important outcomes (eg, PTSD or depression).^28^ Only a handful of studies have explored symptom trajectories jointly: comorbid PTSD and depressive symptom trajectories among US military personnel^29^ and children surviving a natural disaster^30^; comorbid PGD and depressive symptom trajectories among bereaved family caregivers of patients who are terminally ill^31,32^; comorbid PGD, PTSD, and depressive symptom trajectories among people who have experienced disaster-related bereavement^33^; and bereaved adults who lost a loved one from a mixed natural or violent death.^34^ Ignoring co-occurrence of distressed psychological grief reactions may obscure symptom trajectory interactions, hamper differentiation of various psychological presentations, and limit effectiveness of treatments.

Also, methodological insufficiencies regarding timing of assessments limit appropriate modeling of PGD, PTSD, and depressive symptom trajectories. For example, approximately annual,^13,24,30,33^ biennial,^23,24,26^ every 3 years,^15,25,29^ or sparser^16,22^ assessments imprecisely detect changes in symptoms. Short-term assessments within 1 year of loss^12^ neglect long-term bereavement adjustment, and besides, PGD symptoms must persist beyond the first anniversary of the loss to meet the *Diagnostic and Statistical Manual of Mental Disorders* (Fifth Edition, Text Revision) (*DSM-5-TR*) minimal duration criterion.^35^ Finally, assessments conducted only twice^14^ overlook growth patterns^36^ of PGD, PTSD, and depression symptoms after the death. Therefore, the purpose of this study was to simultaneously explore latent trajectories of PGD, PTSD, and depressive symptoms among bereaved family surrogates of individuals who died in an ICU from 6 to 24 months after the death with an appropriate time interval reflecting the PGD duration criterion^35,37^ and to examine the co-occurrence of these trajectories.

## Methods

This cohort study was conducted with approval for human participant research by the Chang Gung Medical Foundation institutional review board. Each family surrogate signed informed consent for participation. This study was reported according to the Strengthening the Reporting of Observational Studies in Epidemiology (STROBE) reporting guideline for cohort studies.

### Design, Setting, and Participants

Data for this study are from a cohort study on associations of quality of end-of-life ICU care with family bereavement outcomes, including symptoms of anxiety, depression,^38^ PTSD, and PGD.^39^ Information on sampling strategy, patient and family characteristics, and study settings are reported elsewhere.^38,39^ Family members responsible for decision-making for patients who are critically ill and at high risk of death (acute physiology and chronic health evaluation APACHE II scores >20) were consecutively recruited in level III medical ICUs of 2 academically affiliated hospitals in Taiwan from January 2018 to March 2020 and followed-up through July 2022.

### Data Collection

Phone interviews assessed surrogate psychological distress at 1, 3, 6, 13, 18, and 24 months after the death of the family member, adhering with the minimum duration criterion for PTSD (≥1 month)^40^ and avoiding the anniversary effect at 12 months after the death. Only data collected starting 6 months after the death were used in this study to accommodate the PGD duration criterion.^37^

### Measures

PGD symptoms were measured by 11 items of the Prolonged Grief Disorder–13 item (PG-13),^37^ including 1 separation distress symptom, 9 cognitive and emotional symptoms, and 1 functional impairment symptom. The 2 dichotomous PG-13 items regarding duration and impairment criteria were excluded because they do not measure grief symptoms. Frequency of symptoms in the preceding month were rated on a 5-point Likert scale (1 indicates never; 5, always). PG-13 scores of 30 or greater indicate PGD^41^; therefore, PG-11 scores of 28 or greater indicate severe PGD symptoms.

PTSD symptoms were measured by the 22-item Impact of Event Scale–Revised (IES-R).^40^ IES-R measures PTSD symptoms on 3 subscales: intrusion, avoidance, and hyperarousal. For each item, the PTSD symptom distress level during the preceding week was evaluated on a Likert scale from 0, indicating not at all to 4, extremely.^40^ IES-R scores of 33 or greater indicate severe PTSD symptoms.^40^

Depressive symptoms were measured by the 7-item depression subscale of the Hospital Anxiety and Depression Scale (HADS-D).^42^ HADS-D total scores range from 0 to 21.^42^ Severe depressive symptoms correspond to scores of 8 or greater.^38,42^

### Statistical Analysis

Latent growth mixture modeling^43,44^ was conducted to simultaneously examine the latent trajectories of PGD, PTSD, and depression symptoms using total PG-11, IES-R, and HADS-D scores using Latent GOLD software version 5.1 (Statistical Innovations). Latent GOLD provides full information maximum likelihood estimates with missing data on PGD, PTSD, and depression symptoms.^45^ We began with a 1-trajectory linear model (including slope and intercept parameters) and increased the number of trajectories for each symptom until the best model emerged (eMethods in Supplement 1).

Co-occurrence of PGD, PTSD, and depressive symptom trajectories was first presented graphically and next identified by joint latent class analysis (JLCA). JLCA systematically groups multiple discrete latent variables into joint patterns that consist of individuals who share a similar combination of PGD, PTSD, and depressive symptom trajectories.^46^ Procedures and criteria for selecting the best model of JLCA are the same as those for latent growth mixture modeling.

Trajectory shapes were decided by significance: the shape was quadratic if the quadratic term was significant (2-sided *P* < .05), and the shape was linear if the quadratic term was not significant and the linear term was. Data were analyzed from August to December 2022.

## Results

### Participant Characteristics

A total of 321 family surrogates of 353 ICU decedents participated in bereavement surveys. Of these, 303 surrogates (94.4%) provided data 6 to 24 months after the death and constituted the study sample; 292 surrogates completed the 6-month survey, 277 surrogates completed the 13-month survey, 275 surrogates completed the 18-month survey, and 261 surrogates completed the survey at 24 months after the death of their family member. Most participants were younger than 56 years (207 participants [68.3%]), female (177 participants [58.4%]), and married (228 participants [75.2%]). A total of 88 surrogates (29.0%) were the patient’s spouse and 166 surrogates (54.8%) were the patient’s adult child. In the year before their family member’s ICU admission, few surrogates reported symptoms of anxiety (8 participants) or depression (3 participants), and surrogates had not been hospitalized for mental health problems.^39^ Participants generally reported low levels of PGD, PTSD, and depressive symptoms across the study period (Table 1).

**Table 1.** Extent of Prolonged Grief Disorder, Posttraumatic Stress Disorder, and Depressive Symptoms

Time since death, mo	Symptoms, mean (SD)		
	Prolonged grief disorder^a^	Posttraumatic stress disorder^b^	Depression^c^
6 (n = 292)	17.15 (6.72)	5.96 (7.11)	4.18 (3.61)
13 (n = 277)	15.12 (5.69)	4.62 (6.43)	3.66 (3.26)
18 (n = 275)	14.53 (5.18)	3.71 (5.94)	3.62 (3.02)
24 (n = 261)	14.03 (4.99)	3.22 (5.16)	3.70 (3.03)

^a^ Range, 1-55; higher score indicates greater severity.

^b^ Range, 0-88; higher score indicates greater severity.

^c^ Range, 0-21; higher score indicates greater severity.

### Latent Trajectories of PGD, PTSD, and Depressive Symptoms at 6 to 24 Months 

Akaike information criteria (AIC), bayesian IC (BIC), and sample-size adjusted BIC (SABIC) decreased, whereas log-likelihood increased consistently (eTable 1 in Supplement 1), suggesting increasing fit with each subsequent combination of numbers of PGD, PTSD, and depressive symptom trajectories. However, AIC, BIC, and SABIC steeply decreased between the joint class model with 3 PGD, 3 PTSD, and 2 depressive symptom trajectories vs the joint class model with 3 PGD, 3 PTSD, and 3 depressive-symptom trajectories. The plots of IC values vs class numbers flattened at the joint class model with 3 PGD, 3 PTSD, and 3 depressive symptom trajectories, suggesting subsequent increases in class numbers were not meaningful (eFigure 1 in Supplement 1). This model also had a higher entropy (indicating a higher certainty in class membership assignment for the 3 psychological symptoms individually and as a whole) (eTable 1 in Supplement 1), was parsimonious, had adequate sample size (Table 2), and was clinically meaningful. PGD, PTSD, and depressive symptom trajectories identified by this model were also confirmed by identifying each symptom trajectory individually. Table 2 details estimated prevalence of identified trajectories and trajectory shapes for PGD, PTSD, and depressive symptoms.

**Table 2.** Estimated Prevalence of Identified Trajectories and Trajectory Shapes (N = 303)^a^

Class	Prevalence	Intercept			Linear			Quadratic		
	No. (%)	β (SE)	*z* Value	*P* value	β (SE)	*z* Value	*P* value	β (SE)	*z* Value	*P* value
**PGD symptom trajectory**										
Resilience	253 (83.5)	16.75 (0.63)	26.49	<.001	−4.52 (1.01)	−4.46	<.001	1.14 (0.38)	3.01	.003
Recovery	36 (11.9)	29.14 (1.70)	17.11	<.001	−10.21 (2.63)	−3.88	<.001	2.55 (0.98)	2.60	.009
Chronic	14 (4.6)	38.42 (2.53	15.22	<.001	−7.28 (4.02)	−1.81	.070	1.52 (1.50	1.02	.310
**PTSD symptom trajectory **										
Resilience	250 (82.5)	6.04 (0.74)	8.21	<.001	−4.39 (1.14)	−3.85	<.001	1.10 (0.42)	2.65	.008
Recovery	41 (13.5)	25.39 (2.43)	10.45	<.001	−14.12 (3.65)	−3.87	<.001	3.10 (1.32)	2.35	.019
Delayed-onset	12 (4.0)	11.36 (3.68)	3.09	.002	26.31 (5.67)	4.64	<.001	−10.43 (2.06)	−5.08	<.001
**Depressive symptom trajectory**										
Resilience	200 (66.0)	2.94 (0.44)	6.73	<.001	−1.79 (0.73)	−2.46	.014	0.69 (0.28)	2.48	.013
Moderate	72 (23.8)	6.86 (0.73)	9.39	<.001	−1.53 (1.18)	−1.30	.190	0.30 (0.45)	0.66	.510
Chronic	31 (10.2)	12.04 (1.05)	11.43	<.001	−2.97 (1.70)	−1.75	.081	0.96 (0.64)	1.50	.130

Abbreviations: PGD, prolonged grief disorder; PTSD, posttraumatic stress disorder.

^a^ Shapes of trajectories were determined by fitting polynomial regressions of 11 items from the Prolonged Grief Disorder–13, Impact of Event Scale–Revised, and 7-item depression subscale of the Hospital Anxiety and Depression Scale scores on linear and quadratic terms.

Among 303 participants, a resilience trajectory predominantly described their PGD (253 participants [83.5%]), PTSD (250 participants [82.5%]), and depressive (200 participants [66.0%]) symptoms. This trajectory had a low intercept, with significant linear and quadratic slopes (Table 2 and Figure). Symptom levels of resilience trajectories significantly decreased and were low overall.

**Figure.**
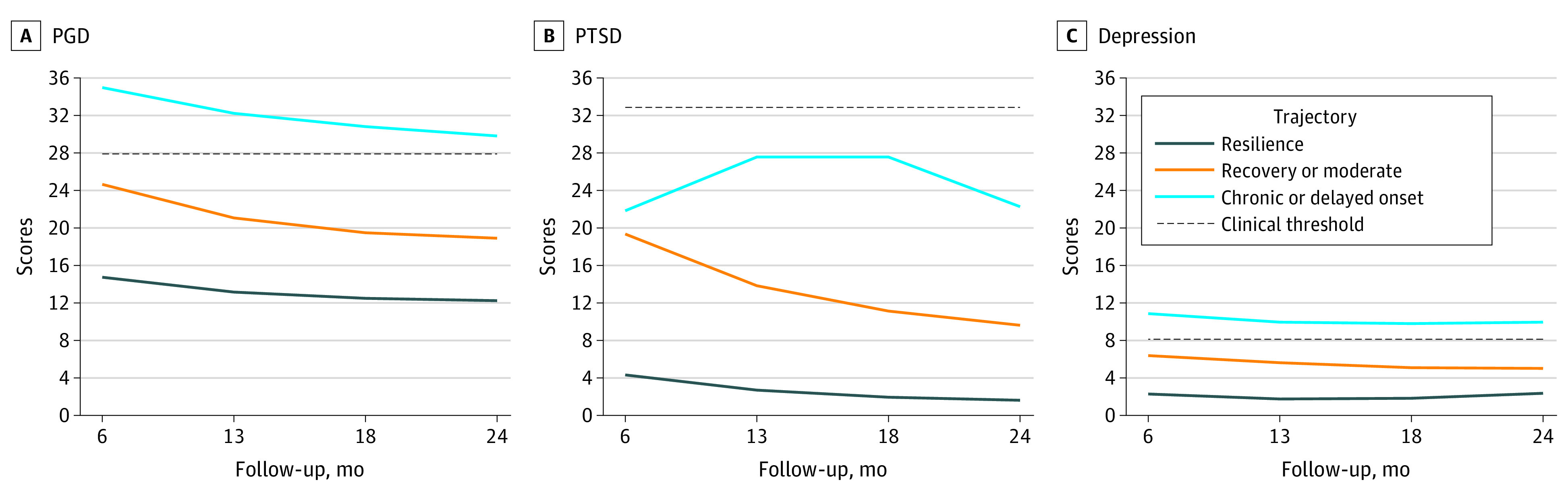
Latent Trajectories of PGD, PTSD, and Depressive Symptoms 6 to 24 Months After Loss The horizontal dashed black lines indicate the threshold for severe symptoms. PGD indicates prolonged grief disorder; PTSD, posttraumatic stress disorder.

A recovery trajectory, the second most descriptive of PGD (36 participants [11.9%]) and PTSD (41 participants [13.5%]) symptoms, had a moderately high intercept, with significant linear and quadratic slopes (Table 2 and Figure). A moderate trajectory, the second most descriptive for depressive symptoms (72 participants [23.8%]), had a moderately high subthreshold intercept, with insignificant linear and quadratic slopes. The observed quadratic shapes for the recovery PGD and PTSD symptom trajectories indicate general patterns of steep initial decline followed by slowed resolution. Severity of depressive symptoms remained moderate throughout the 24 months of bereavement follow-up.

A chronic trajectory, identified for PGD symptoms in 14 participants (4.6%) and depressive symptoms in 31 participants (10.2%), had a high intercept with insignificant linear and quadratic slopes (Table 2 and Figure). A unique delayed-onset trajectory was identified for PTSD symptoms in 12 participants (4.0%) and had a moderate-to-high symptom level that increased significantly from 6 to 13 months after the death, plateaued between 13 to 18 months, subsided quickly thereafter (shown by the significant quadratic slope), but remained far above the recovery and resilience trajectories at 24 months after the death (Table 2 and Figure).

### Joint Patterns of PGD, PTSD, and Depressive Symptom Trajectories

Table 3 shows the co-occurrence of PGD, PTSD, and depressive symptom trajectories during the course of follow-up. Overall, trajectories cooccurred for 228 participants (75.2%). However, multiple patterns were discordant. The most prevalent discordant patterns were co-occurrence of resilience (48 participants [15.8%]) or recovery (14 participants [4.6%]) trajectories for PGD and PTSD symptoms combined with a higher level of depressive symptoms trajectory (Table 3; eFigure 2 in Supplement 1).

**Table 3.** Cross-Tabulation of Co-Occurrence of PGD-, PTSD-, and Depressive-Symptom Trajectories (N = 303)

Depressive symptom trajectory	PGD symptom trajectory	Participants by PTSD symptom trajectory, No.			Total, No.
		Resilience	Recovery	Delayed-onset	
Resilience	Resilience	200^a^	0	0	200
Moderate	Resilience	48^b^	4	0	52
	Recovery	0	18^a^	1	19
	Chronic	0	1	0	1
Chronic	Resilience	0	1	0	1
	Recovery	2	14^b^	1	17
	Chronic	0	3	10^a^	13
Total	NA	250	41	12	303

Abbreviations: NA, not applicable; PGD, prolonged grief disorder; PTSD, posttraumatic stress disorder.

^a^ Indicates the co-occurrence of same patterns of PGD, PTSD, and depressive symptom trajectories.

^b^ Indicates the most prevalent patterns of disconcordance of PGD, PTSD, and depressive symptom trajectories.

Our JLCA made it possible to summarize the 27 (3 × 3 × 3) possible combinations of symptom trajectories. The 3-class solution was selected as the most optimal joint pattern by the best combination of ICs (eTable 2 and eFigure 3 in Supplement 1), adequate class sizes, parsimony, and clinical meaningfulness. Based on the conditional probabilities (eMethods in Supplement 1) of each symptom trajectory, these 3 joint patterns were named *resilient* (247 participants [81.5%]), *recovered* (43 participants [14.1%]), and *distressed* (14 participants [4.5%]) (Table 4).

**Table 4.** Conditional Probabilities and Prevalence of the 3-Class Solution of Joint Patterns of PGD, PTSD, and Depressive Symptom Trajectories (N = 303)

Symptoms	Trajectories	Conditional probability		
		Resilient pattern (n = 247)	Recovered pattern (n = 43)	Distressed pattern (n = 14)
PGD	Resilience	0.999	0.105	0.010
	Recovery	0.001	0.854	0.069
	Chronic	0.001	0.042	0.921
PTSD	Resilience	0.999	0.074	0.009
	Recovery	0.001	0.890	0.202
	Delayed-onset	0.000	0.036	0.789
Depression	Resilience	0.804	0.003	0.008
	Moderate	0.196	0.588	0.012
	Chronic	0.001	0.409	0.980

Abbreviations: PGD, prolonged grief disorder; PTSD, posttraumatic stress disorder.

Participants in the resilient pattern had extremely high conditional probabilities of being in the resilience trajectory for all 3 psychological symptoms (PGD, 0.999; PTSD, 0.999; depressive, 0.804) (Table 4). Surrogates in this pattern showed minimal symptom distress across the 3 psychological symptoms.

Participants in the recovered pattern predominantly belonged to the recovery trajectory for PGD (conditional probability, 0.854) and PTSD (conditional probability, 0.890) symptoms and had a high conditional probability (0.588) of being in the moderate trajectory for depressive symptoms (Table 4). Surrogates in this group experienced moderately high PGD, PTSD, and depressive symptoms at 6 months after the death of their family member, and their PGD and PTSD symptoms subsided by 24 months. Still, their moderate depressive symptoms persisted throughout the second bereavement year (Figure).

Participants in the distressed pattern had high conditional probabilities of being in the chronic trajectory for PGD (conditional probability, 0.921) and depressive (conditional probability, 0.980) symptoms (Table 4) but with a predominant conditional probability (0.789) of being in the delayed-onset trajectory for PTSD symptoms. Surrogates in this group consistently experienced high distress from PGD and depressive symptoms throughout the first 2 bereavement years, whereas high PTSD symptoms increased, subsided, but remained high by the end of the second bereavement year (Figure).

## Discussion

This cohort study identified 3 symptom trajectories each for PGD, PTSD, and depressive symptoms for family surrogates of patients who died in the ICU, 6 to 24 months after the death. Trajectory comparisons between our study and the 2 existing studies^33,34^ are in eTable 3 in Supplement 1. Briefly, all studies identified a comparable resilience trajectory most common for all symptoms. Djelantik and colleagues^34^ reported substantially lower prevalence for PGD resilience, with measurements primarily during the first bereavement year. Our chronic trajectory for PGD and depressive symptoms was identified across all symptoms in the existing studies.^33,34^ Notably, bereaved individuals in our study, whose deceased family member predominantly had a natural cause of death, reported persistence or decrease^34^ in elevated symptoms, whereas in the study by Lenferink et al^33^ bereaved individuals whose family member died in a disaster experienced increasing symptoms.

Our findings illuminate discrepancies: recovery described PGD and PTSD symptoms in our study, whereas Lenferink et al^33^ found recovery trajectories predominant in PTSD and depressive symptoms and Djelantik et al^34^ found a recovery trajectory was predominant in PGD symptoms only. Lenferink and colleagues^33^ suggested PGD symptoms are more stable, but other studies have identified recovery of PGD symptoms within 3 to 25 months after a death.^13,14,34^ Perhaps PGD recovery could not be detected by the infrequent yearly assessment.^33^ Our recovery trajectory was also significantly less severe than the chronic trajectory from 6 to 24 months after the death, differing from the study by Djelantik and colleagues^34^ but consistent with other studies.^12,13,15,16^

The delayed-onset trajectory identified for PTSD symptoms in our study was unique to our study. Beyond bereavement literature, a relapse or delayed-onset trajectory was reported among US military personnel^29^ and children surviving a natural disaster^30^ for PTSD and depressive symptoms, and among adult spouses of veterans who had experienced trauma^21^ for PTSD symptoms. Regarding the bereaved individuals, researchers have suggested that delayed grief may occur infrequently, if at all.^10^ However, a systematic review of psychological responses to major life stressors, including bereavement, reported the synthesized prevalence of delayed-onset trajectory as 8.9% (95% CI, 5.3%-13.3%).^5^ We observed an in-between prevalence (4.0%) of delayed-onset PTSD symptom trajectory for bereaved surrogates of patients who died in an ICU. Indeed, a 2022 systematic review^47^ found a delayed PTSD trajectory in 29 of 42 included study populations. Explanations offered by the systematic review by Bonde et al^47^ for why a delayed PTSD trajectory may not be observed are applicable to the 2 prior bereavement symptom trajectory studies^33,34^: (1) sample size may be too small to detect an infrequent trajectory,^33^ and (2) the follow-up period may be too short.^34^ Our identified delayed-onset PTSD symptom trajectory needs to be validated by further research with a larger sample and longer follow-ups.

Approximately three-quarters (75.2%) of study participants had consistent symptom trajectories for PGD, PTSD, and depressive symptoms, but multiple discordant combinations were evident. Our results suggest that resilience in 1 domain often coexisted with deficits in other important domains.^28^ Our JLCA identified 3 joint patterns: resilient, recovered, and distressed patterns.

Participants in the resilient pattern exhibited mild symptoms at 6 months after the death of their family member that subsided quickly without developing into any clinically relevant psychological disorder from 6 to 24 months after the death.^24,48,49^ Our results confirm that resilience is the modal pattern of reaction to major life stressors, including bereavement,^5^ with some exceptions. Previous studies have found that bereaved individuals who were the spouse of the deceased and who were older were infrequently in the resilient pattern, identified by absence of clinically severe depressive symptoms or significant emotional or social loneliness^25^ or by lower depression, hopelessness, and loneliness, higher life satisfaction, and better subjective health.^48^ Similarly, a study in China by Zhou et al^50^ reported that bereaved parents who lost their only child were not frequently identified as resilient by physical health and psychological outcomes (PGD, PTSD, depressive symptoms, posttraumatic growth).

The substantially higher prevalence of the resilient pattern in our study may be attributed to several social and cultural factors. First, bereaved individuals in Taiwan are in a more collectivist and family-centered culture than individualist European or US cultures. Taiwanese family members tend to provide emotional, practical, and financial support^51^ for the bereaved, thereby increasing resilience.

Second, cultural grieving traditions also contribute to our observed high prevalence of the resilient pattern. In Taiwanese culture, 3 funeral ceremonies periodically scheduled within 100 days of death facilitate public expression of sorrow and grief. After these 100 days, public displays of grief are not the social norm,^52^ thereby potentially increasing the likelihood that our participants remained resilient at the first 6-month follow-up after the death of their family member compared with samples among Western cultures.^25,48^

Lastly, our higher prevalence of the resilient pattern than that reported for bereaved parents in China^50^ may be attributed to the context of losses. Bereavement is one of the most difficult life experience for many people; in China and Taiwan, proverbs say, “the most eternal hardship is death,” and “the three biggest misfortunes in life are to be fatherless in youth, widowed in middle age, and childless in old age.” Losing a child is an especially difficult bereavement experience not only because it violates the expectation that children should grow up healthy and outlive their parents,^53^ but in Chinese and Taiwanese culture, it also defies the cultural value of filial piety. Chinese culture recognizes 3 forms of unfilial conduct, of which the worst is to have no descendants. Therefore, for Chinese parents, losing an only child may predispose them to severe grief-related psychological distress, barring them from the resilient joint pattern.

Participants in the recovered pattern reported moderate PGD and PTSD symptoms 6 months after the death of their family member and experienced significant reductions in these grief symptoms over time, as well as persistent subthreshold depressive symptoms (HADS-D score, <8). These participants may contend with the loss, gradually recover,^54^ and return to their baseline psychological distress status within the first 2 years of bereavement.

We found that 4.5% of participants fell into the distressed pattern, with chronic, high-level PGD, PTSD, and depressive-symptom trajectories. The proportion of bereaved individuals who had the poorest functioning has been reported as 7% for bereaved spouses in older age experiencing depressive symptoms, hopelessness, and loneliness^48^; 8.4% for Chinese parents who lost an only child and faced high levels of physical impairment, negative psychological outcomes, and lower levels of posttraumatic growth^50^; 12.5% for bereaved individuals in older age facing depressive symptoms, emotional loneliness, and social loneliness^25^; and 15.4% for bereaved family members in PGD, PTSD, and depressive symptom trajectories.^34^ Our explanations for the higher prevalence rate for the resilient pattern can be applied to why our participants had the lowest probability for the distressed pattern. Although the prevalence was low, existence of this distressed pattern highlights the need for early screening, identification, and effectively tailoring interventions to avoid prolonged severe distress.

### Limitations

This study has some limitations. Our study findings may not be applicable to international populations beyond the sampled hospitals. We used self-reports by screening rather than diagnostic tools to assess PGD, PTSD, and depression symptoms, which may overestimate the severity of symptoms and are subject to both recall and reporting bias that could affect the findings. PGD was assessed by 11 items from the PG-13,^37^ not the most updated criteria like the PG-13-Revised^35^ or *International Classification of Diseases, 11th Revision *(*ICD-11*) PGD criteria. Our findings cannot robustly generalize to grief reactions from less than 6 months or more than 24 months after the death of a family member. Additionally, drawing from the integrative framework of factors associated with bereavement outcomes,^54^ including intrapersonal (eg, symptoms of PGD, PTSD, and depression prior to their family member’s ICU admission), interpersonal, bereavement-related stressors, and death circumstances, in estimating membership in distinct trajectories or joint patterns of PGD, PTSD, and depressive-symptom trajectories, especially for the most worrisome distressed pattern, have not yet been explored.

## Conclusions

In this cohort study, we identified resilient, recovered, and distressed conjoint patterns of PGD, PTSD, and depressive symptom trajectories that may have clinical utility to inform the timing of early screening, identification, and intervention. Among bereaved surrogates of patients who die in an ICU, a significant portion experienced persistent elevation in combined symptoms of PGD, PTSD, and depression from 6 to 24 months after the death of their family member, underscoring the importance of prevention, early screening and identification, and effective treatments to target this population at high risk of adverse outcomes.

## Notes

Supplement 1. **eMethods.** Processes and Criteria for Identification of PGD, PTSD, and Depressive Symptom Trajectories and Conjoint Patterns of PGD, PTSD, and Depressive Symptom Trajectories **eTable 1.** Model Fit Indices for Class Solutions With Increasing Number of PGD, PTSD, and Depressive Symptom Trajectories **eFigure 1.** Model Fit Figures for Class Solutions With Increasing Number of PGD, PTSD, and Depressive Symptom Trajectories **eFigure 2.** Co-Occurrence of PGD, PTSD, and Depressive Symptom Trajectories **eTable 2.** Model Fit Indices for Different Joint Pattern Solutions of PGD, PTSD, and Depressive Symptom Trajectories **eFigure 3.** Model Fit Figures for Different Joint Pattern Solutions of PGD, PTSD, and Depressive Symptom Trajectories **eTable 3.** Comparisons of Identified PGD, PTSD, and Depressive Symptom Trajectories Across the 2 Existing Studies and the Current Study

Supplement 2.
Data Sharing Statement

## References

[1] Weissman GE, Kerlin MP, Yuan Y, . Population trends in intensive care unit admissions in the United States among Medicare beneficiaries, 2006-2015. Ann Intern Med. 2019;170(3):213-215. doi:10.7326/M18-1425 30326008 PMC6467736

[2] Teno JM, Gozalo P, Trivedi AN, . Site of death, place of care, and health care transitions among US Medicare beneficiaries, 2000-2015. JAMA. 2018;320(3):264-271. doi:10.1001/jama.2018.8981 29946682 PMC6076888

[3] Armstrong RA, Kane AD, Cook TM. Outcomes from intensive care in patients with COVID-19: a systematic review and meta-analysis of observational studies. Anaesthesia. 2020;75(10):1340-1349. doi:10.1111/anae.15201 32602561

[4] Holmes TH, Rahe RH. The social readjustment rating scale. J Psychosom Res. 1967;11(2):213-218. doi:10.1016/0022-3999(67)90010-4 6059863

[5] Galatzer-Levy IR, Huang SH, Bonanno GA. Trajectories of resilience and dysfunction following potential trauma: a review and statistical evaluation. Clin Psychol Rev. 2018;63:41-55. doi:10.1016/j.cpr.2018.05.008 29902711

[6] Infurna FJ, Luthar SS. Resilience to major life stressors is not as common as thought. Perspect Psychol Sci. 2016;11(2):175-194. doi:10.1177/1745691615621271 26993272 PMC4800830

[7] Kentish-Barnes N, Chaize M, Seegers V, . Complicated grief after death of a relative in the intensive care unit. Eur Respir J. 2015;45(5):1341-1352. doi:10.1183/09031936.00160014 25614168

[8] Azoulay E, Pochard F, Kentish-Barnes N, ; FAMIREA Study Group. Risk of post-traumatic stress symptoms in family members of intensive care unit patients. Am J Respir Crit Care Med. 2005;171(9):987-994. doi:10.1164/rccm.200409-1295OC 15665319

[9] Kross EK, Engelberg RA, Gries CJ, Nielsen EL, Zatzick D, Curtis JR. ICU care associated with symptoms of depression and posttraumatic stress disorder among family members of patients who die in the ICU. Chest. 2011;139(4):795-801. doi:10.1378/chest.10-0652 20829335 PMC3071273

[10] Bonanno GA, Diminich ED. Annual research review: positive adjustment to adversity–trajectories of minimal-impact resilience and emergent resilience. J Child Psychol Psychiatry. 2013;54(4):378-401. doi:10.1111/jcpp.12021 23215790 PMC3606676

[11] Seiler A, von Känel R, Slavich GM. The psychobiology of bereavement and health: a conceptual review from the perspective of social signal transduction theory of depression. Front Psychiatry. 2020;11:565239. doi:10.3389/fpsyt.2020.565239 33343412 PMC7744468

[12] Lundorff M, Bonanno GA, Johannsen M, O’Connor M. Are there gender differences in prolonged grief trajectories: a registry-sampled cohort study. J Psychiatr Res. 2020;129:168-175. doi:10.1016/j.jpsychires.2020.06.030 32739617

[13] Bonanno GA, Malgaroli M. Trajectories of grief: comparing symptoms from the *DSM-5* and *ICD-11* diagnoses. Depress Anxiety. 2020;37(1):17-25. doi:10.1002/da.22902 31012187

[14] Djelantik AAAMJ, Smid GE, Kleber RJ, Boelen PA. Early indicators of problematic grief trajectories following bereavement. Eur J Psychotraumatol. 2018;8(suppl 6):1423825. 29372008 10.1080/20008198.2018.1423825 PMC5774421

[15] Sveen J, Bergh Johannesson K, Cernvall M, Arnberg FK. Trajectories of prolonged grief one to six years after a natural disaster. PLoS One. 2018;13(12):e0209757. doi:10.1371/journal.pone.0209757 30576369 PMC6303052

[16] Kristensen P, Dyregrov K, Gjestad R. Different trajectories of prolonged grief in bereaved family members after terror. Front Psychiatry. 2020;11:545368. doi:10.3389/fpsyt.2020.545368 33192660 PMC7591785

[17] Smith KV, Ehlers A. Cognitive predictors of grief trajectories in the first months of loss: a latent growth mixture model. J Consult Clin Psychol. 2020;88(2):93-105. doi:10.1037/ccp0000438 31556649 PMC6939605

[18] Ambler M, Rhoads S, Peterson R, . One year later: family members of patients with COVID-19 experience persistent symptoms of posttraumatic stress disorder. Ann Am Thorac Soc. 2023;20(5):713-720. doi:10.1513/AnnalsATS.202209-793OC 36508292 PMC10174132

[19] Wendlandt B, Pongracz L, Lin FC, . Posttraumatic stress symptom trajectories in family caregivers of patients with acute cardiorespiratory failure. JAMA Netw Open. 2023;6(4):e237448. doi:10.1001/jamanetworkopen.2023.7448 37027154 PMC10082401

[20] Wendlandt B, Chen YT, Lin FC, . Posttraumatic stress disorder symptom trajectories in ICU family caregivers. Crit Care Explor. 2021;3(4):e0409. doi:10.1097/CCE.0000000000000409 33912839 PMC8078333

[21] Zerach G, Horesh D, Solomon Z. Secondary posttraumatic stress symptom trajectories and perceived health among spouses of war veterans: a 12-year longitudinal study. Psychol Health. 2022;37(6):675-691. doi:10.1080/08870446.2021.1879807 33626993

[22] Lotterman JH, Bonanno GA, Galatzer-Levy I. The heterogeneity of long-term grief reactions. J Affect Disord. 2014;167:12-19. doi:10.1016/j.jad.2014.05.048 25082108

[23] Maccallum F, Galatzer-Levy IR, Bonanno GA. Trajectories of depression following spousal and child bereavement: a comparison of the heterogeneity in outcomes. J Psychiatr Res. 2015;69:72-79. doi:10.1016/j.jpsychires.2015.07.017 26343597

[24] Infurna FJ, Grimm KJ. The use of growth mixture modeling for studying resilience to major life stressors in adulthood and old age: lessons for class size and identification and model selection. J Gerontol B Psychol Sci Soc Sci. 2017;73(1):148-159. doi:10.1093/geronb/gbx019 28329850 PMC5927099

[25] Szabó Á, Kok AAL, Beekman ATF, Huisman M. Longitudinal examination of emotional functioning in older adults after spousal bereavement. J Gerontol B Psychol Sci Soc Sci. 2020;75(8):1668-1678. doi:10.1093/geronb/gbz039 30953058

[26] Chen C, Chow AYM, Tang S. Trajectories of depression symptoms in Chinese elderly during widowhood: a secondary analysis. Aging Ment Health. 2020;24(8):1254-1262. doi:10.1080/13607863.2019.1603285 30983380

[27] Komischke-Konnerup KB, Zachariae R, Johannsen M, Nielsen LD, O’Connor M. Co-occurrence of prolonged grief symptoms and symptoms of depression, anxiety, and posttraumatic stress in bereaved adults: a systematic review and meta-analysis. J Affect Disord Rep. 2021;4:100140. doi:10.1016/j.jadr.2021.100140

[28] Infurna FJ, Luthar SS. The multidimensional nature of resilience to spousal loss. J Pers Soc Psychol. 2017;112(6):926-947. doi:10.1037/pspp0000095 27399253 PMC5226923

[29] Armenta RF, Walter KH, Geronimo-Hara TR, Porter B, Stander VA, LeardMann CA; Millennium Cohort Study Team. Longitudinal trajectories of comorbid PTSD and depression symptoms among U.S. service members and veterans. BMC Psychiatry. 2019;19(1):396. doi:10.1186/s12888-019-2375-1 31836015 PMC6911296

[30] Liang Y, Zhou Y, Liu Z. Consistencies and differences in posttraumatic stress disorder and depression trajectories from the Wenchuan earthquake among children over a 4-year period. J Affect Disord. 2021;279:9-16. doi:10.1016/j.jad.2020.09.107 33035749

[31] Nam I. Trajectories of complicated grief. Eur J Psychiatry. 2015;29(3):173-182. doi:10.4321/S0213-61632015000300002

[32] Wen FH, Chou WC, Shen WC, Tang ST. Distinctiveness of prolonged-grief-disorder- and depressive-symptom trajectories in the first 2 years of bereavement for family caregivers of terminally ill cancer patients. Psychooncology. 2020;29(10):1524-1532. doi:10.1002/pon.5441 32539210

[33] Lenferink LIM, Nickerson A, de Keijser J, Smid GE, Boelen PA. Trajectories of grief, depression, and posttraumatic stress in disaster-bereaved people. Depress Anxiety. 2020;37(1):35-44. doi:10.1002/da.22850 30339302 PMC7028032

[34] Djelantik AAAMJ, Robinaugh DJ, Boelen PA. The course of symptoms in the first 27 months following bereavement: a latent trajectory analysis of prolonged grief, posttraumatic stress, and depression. Psychiatry Res. 2022;311:114472. doi:10.1016/j.psychres.2022.114472 35248806 PMC9159380

[35] Prigerson HG, Boelen PA, Xu J, Smith KV, Maciejewski PK. Validation of the new *DSM-5-TR* criteria for prolonged grief disorder and the PG-13-Revised (PG-13-R) scale. World Psychiatry. 2021;20(1):96-106. doi:10.1002/wps.20823 33432758 PMC7801836

[36] van de Schoot R. Latent trajectory studies: the basics, how to interpret the results, and what to report. Eur J Psychotraumatol. 2015;6:27514. doi:10.3402/ejpt.v6.27514 25735413 PMC4348410

[37] Prigerson HG, Horowitz MJ, Jacobs SC, . Prolonged grief disorder: psychometric validation of criteria proposed for *DSM-V* and *ICD-11*. PLoS Med. 2009;6(8):e1000121. doi:10.1371/journal.pmed.1000121 19652695 PMC2711304

[38] Tang ST, Huang CC, Hu TH, . End-of-life-care quality in ICUs is associated with family surrogates’ severe anxiety and depressive symptoms during their first 6 months of bereavement. Crit Care Med. 2021;49(1):27-37. doi:10.1097/CCM.0000000000004703 33116053

[39] Tang ST, Huang CC, Hu TH, Chou WC, Chuang LP, Chiang MC. Course and predictors of posttraumatic stress-related symptoms among family members of deceased ICU patients during the first year of bereavement. Crit Care. 2021;25(1):282. doi:10.1186/s13054-021-03719-x 34353352 PMC8340476

[40] Weiss DS, Marmar CR. The impact of event scale—revised. In: Wilson JP, Keane TM, eds. Assessing Psychological Trauma and PTSD. Guilford Press; 1997:399-411.

[41] Kentish-Barnes N, Chevret S, Valade S, . A three-step support strategy for relatives of patients dying in the intensive care unit: a cluster randomised trial. Lancet. 2022;399(10325):656-664. doi:10.1016/S0140-6736(21)02176-0 35065008

[42] Zigmond AS, Snaith RP. The hospital anxiety and depression scale. Acta Psychiatr Scand. 1983;67(6):361-370. doi:10.1111/j.1600-0447.1983.tb09716.x 6880820

[43] Muthén B. Latent variable analysis: growth mixture modeling and related techniques for longitudinal data. In: Kaplan D, ed. The Sage Handbook of Quantitative Methodology for the Social Sciences. Sage; 2004:345-369. doi:10.4135/9781412986311.n19

[44] Nylund KL, Asparouhov T, Muthén B. Deciding on the number of classes in latent class analysis and growth mixture modeling. a Monte Carlo simulation study. Struct Equ Modeling. 2007;14(4):535-569. doi:10.1080/10705510701575396

[45] Vermunt JK, Magidson J. Technical Guide for Latent GOLD 5.1: Basic, Advanced, and Syntax. Statistical Innovations Inc; 2016.

[46] Jeon S, Lee J, Anthony JC, Chung H. Latent class analysis for multiple discrete latent variables: a study on the association between violent behavior drug-using behaviors. Struct Equ Modeling. 2017;24(6):911-925. doi:10.1080/10705511.2017.1340844 30828241 PMC6395048

[47] Bonde JPE, Jensen JH, Smid GE, . Time course of symptoms in posttraumatic stress disorder with delayed expression: a systematic review. Acta Psychiatr Scand. 2022;145(2):116-131. doi:10.1111/acps.13372 34523121 PMC9293462

[48] Spahni S, Morselli D, Perrig-Chiello P, Bennett KM. Patterns of psychological adaptation to spousal bereavement in old age. Gerontology. 2015;61(5):456-468. doi:10.1159/000371444 25720748

[49] Vella SLC, Pai NB. A theoretical review of psychological resilience: defining resilience and resilience research over the decades. Arch Med Health Sci. 2019;7(2):233-239.

[50] Zhou N, Yu W, Huang H, . Latent profiles of physical and psychological outcomes of bereaved parents in China who lost their only child. Eur J Psychotraumatol. 2018;9(1):1544026. doi:10.1080/20008198.2018.1544026 30479701 PMC6249556

[51] Chow AY, Chan CL, Ho SM. Social sharing of bereavement experience by Chinese bereaved persons in Hong Kong. Death Stud. 2007;31(7):601-618. doi:10.1080/07481180701405105 17847573

[52] Tsai PJ. Pattern of grief expression in Chinese families. Taiwan Counseling Quarterly. 2012;4(1):16-38.

[53] Zetumer S, Young I, Shear MK, . The impact of losing a child on the clinical presentation of complicated grief. J Affect Disord. 2015;170:15-21. doi:10.1016/j.jad.2014.08.021 25217759 PMC4253869

[54] Stroebe MS, Folkman S, Hansson RO, Schut H. The prediction of bereavement outcome: development of an integrative risk factor framework. Soc Sci Med. 2006;63(9):2440-2451. doi:10.1016/j.socscimed.2006.06.012 16875769

