# Supplementary material for: Comorbid Prolonged Grief, PTSD, and Depression Trajectories for Bereaved Family Surrogates
Source: JAMA Netw Open. 2023 Nov 10;6(11):e2342675. doi: 10.1001/jamanetworkopen.2023.42675 (PMC10638651; doi:10.1001/jamanetworkopen.2023.42675)
Supplement: Supplement 1. — eMethods. Processes and Criteria for Identification of PGD, PTSD, and Depressive Symptom Trajectories and Conjoint Patterns of PGD, PTSD, and Depressive Symptom Trajectories eTable 1. Model Fit Indices for Class Solutions With Increasing Number of PGD, PTSD, and Depressive Symptom Trajectories eFigure 1. Model Fit Figures for Class Solutions With Increasing Number of PGD, PTSD, and Depressive Symptom Trajectories eFigure 2. Co-Occurrence of PGD, PTSD, and Depressive Symptom Trajectories eTable 2. Model Fit Indices for Different Joint Pattern Solutions of PGD, PTSD, and Depressive Symptom Trajectories eFigure 3. Model Fit Figures for Different Joint Pattern Solutions of PGD, PTSD, and Depressive Symptom Trajectories eTable 3. Comparisons of Identified PGD, PTSD, and Depressive Symptom Trajectories Across the 2 Existing Studies and the Current Study [file jamanetwopen-e2342675-s001.pdf]

# Supplemental Online Content

Wen F, Prigerson HG, Chou W, et al. Comorbid prolonged grief, PTSD, and depression trajectories for bereaved family surrogates. *JAMA Netw Open*. 2023;6(11):e2342675. doi:10.1001/jamanetworkopen.2023.42675

**eMethods.** Processes and Criteria for Identification of PGD, PTSD, and Depressive Symptom Trajectories and Conjoint Patterns of PGD, PTSD, and Depressive Symptom Trajectories
**eTable 1.** Model Fit Indices for Class Solutions With Increasing Number of PGD, PTSD, and Depressive Symptom Trajectories
**eFigure 1.** Model Fit Figures for Class Solutions With Increasing Number of PGD, PTSD, and Depressive Symptom Trajectories
**eFigure 2.** Co-Occurrence of PGD, PTSD, and Depressive Symptom Trajectories
**eTable 2.** Model Fit Indices for Different Joint Pattern Solutions of PGD, PTSD, and Depressive Symptom Trajectories
**eFigure 3.** Model Fit Figures for Different Joint Pattern Solutions of PGD, PTSD, and Depressive Symptom Trajectories
**eTable 3**. Comparisons of Identified PGD, PTSD, and Depressive Symptom Trajectories Across the 2 Existing Studies and the Current Study

This supplemental material has been provided by the authors to give readers additional information about their work.

© 2023 Wen F et al. *JAMA Network Open.*

**eMethods 1. Processes and criteria for identification of PGD-, PTSD-, and depressive-symptom trajectories and conjoint patterns of PGD-, PTSD-, and depressive-symptom trajectories**

Latent growth mixture modeling (LGMM)[1,2] was conducted to simultaneously examine the latent trajectories of PGD, PTSD, and depression symptoms using total PG-11, IES-R, and HADS-D scores by Latent Gold. Latent GOLD provides full information maximum likelihood (FIML) estimates with missing data on PGD, PTSD, and depression symptoms.[3] Missing values are not replaced or imputed, but the missing data is handled by a FIML estimation. By using a FIML estimation approach, all available data were used to estimate each of the individual symptom trajectories (i.e., PGD-, PTSD, and depressive-symptom trajectories). Multiple imputation and FIML have been demonstrated to obtain similar results when outcome data are missing and when the same information is incorporated in a multiple imputation model as in a FIML estimation.[4]

We began with a 1-trajectory linear model (including slope and intercept parameters) and increased the number of trajectories for each symptom until the best model emerged. Best model was selected based on model fit indices, including several information criteria (IC), i.e., the highest log-likelihood (LL), smallest Akaike (AIC), Bayesian (BIC), and sample-size adjusted Bayesian (SABIC), higher entropy (a measure of certainty in class membership assignment, ranging between 0 and 1), and smallest class with a size no less than 2% of the total sample.[1,5] Whereas the BIC is recognized as the best of the IC, the plateau in plots of IC values vs class number suggests the increase in class number is not meaningful.[1] These criteria, parsimony, and the clinical meaningfulness of the latent-trajectory identification were factored into deciding the optimal number of trajectories.

With best fitting model selected, we investigated the proper shape of each identified trajectory (i.e., linear or quadratic) by fitting polynomial regressions of total PG-11, IES-R, and HADS-D scores on linear and quadratic terms based on surrogates in each grief-related-symptom trajectory.[1,5] Trajectory shapes were decided by significance: shape was quadratic if the quadratic term was significant ($p \leq .05$), and shape was linear if the quadratic term was not significant and linear term was.

Co-occurrence of PGD-, PTSD-, and depressive-symptom trajectories was first presented graphically and next identified by joint latent class analysis (JLCA). JLCA systematically groups multiple discrete latent variables into joint patterns that consist of individuals who share a similar combination of PGD-, PTSD-, and depressive-symptom trajectories.[6] Procedures and criteria for selecting the best model of JLCA are the same as those for LGMM. The distribution function for the conditional probability in each identified pattern (as shown on Table 3) reflects

© 2023 Wen F et al. *JAMA Network Open.*

the estimated probability that bereaved surrogates in a pattern belonged to each individual PGD-, PTSD- and depressive-symptom trajectory.[6]

References:

1. Muthén B. Latent variable analysis: Growth mixture modeling and related techniques for longitudinal data. In: Kaplan D, editor. *The Sage handbook of quantitative methodology for the social sciences*. Thousand Oaks, CA: Sage; 2004. pp. 345–369.
2. van de Schoot R. Latent trajectory studies: the basics, how to interpret the results, and what to report. Eur. J. Psychotraumatol. 2015;6:27514.
3. Vermunt JK, Magidson J. *Technical Guide for Latent GOLD 5.1: Basic, Advanced, and Syntax*. Belmont, MA: Statistical Innovations Inc. 2016.
4. Collins LM, Schafer JL, Kam CM. A comparison of inclusive and restrictive strategies in modern missing data procedures. Psychol Methods. 2001;6(4):330-351.
5. Nylund KL, Asparouhov T, Muthén B. Deciding on the number of classes in latent class analysis and growth mixture modeling. A Monte Carlo simulation study. Struct Equ Modeling, 2007; 14, 535–569.
6. Jeon S, Lee J, Anthony JC, Chung H. Latent class analysis for multiple discrete latent variables: A study on the association between violent behavior drug-using behaviors. Struct Equ Modeling. 2017;24(6):911-925.

© 2023 Wen F et al. *JAMA Network Open.*

**eTable 1. Model fit indices for class solutions with increasing number of PGD-, PTSD-, and depressive-symptom trajectories**

| Model[a] | Log-likelihood | BIC[b] | AIC[b] | SABIC[b] | # parameter | Entropy | Individual entropy | | |
|---|---|---|---|---|---|---|---|---|---|
| | | | | | | | PGD | PTSD | depression |
| 222 | -8720.0889 | 17628.7309 | 17506.1778 | 17524.0723 | 33 | 0.615 | 0.971 | 0.991 | 0.903 |
| 223 | -8611.7671 | 17434.9424 | 17297.5343 | 17317.5978 | 37 | 0.730 | 0.971 | 0.991 | 0.860 |
| 232 | -8569.5705 | 17350.5490 | 17213.1409 | 17233.2045 | 37 | 0.615 | 0.971 | 0.965 | 0.903 |
| 322 | -8578.1514 | 17367.7108 | 17230.3027 | 17250.3663 | 37 | 0.615 | 0.936 | 0.991 | 0.903 |
| 233 | -8461.2487 | 17156.7605 | 17004.4974 | 17026.7300 | 41 | 0.730 | 0.971 | 0.965 | 0.860 |
| 323 | -8469.8296 | 17173.9223 | 17021.6592 | 17043.8918 | 41 | 0.730 | 0.933 | 0.993 | 0.858 |
| 332 | -8427.6329 | 17089.5289 | 16937.2659 | 16959.4984 | 41 | 0.615 | 0.936 | 0.965 | 0.903 |
| 333 | -8319.3112 | 16895.7404 | 16728.6224 | 16753.0240 | 45 | **0.730** | **0.936** | **0.965** | **0.860** |
| 334 | -8289.0242 | 16858.0213 | 16676.0484 | 16702.6190 | 49 | 0.780 | 0.936 | 0.965 | 0.772 |
| 343 | -8244.4608 | 16768.8945 | 16586.9216 | 16613.4922 | 49 | 0.730 | 0.936 | 0.917 | 0.860 |
| 433 | -8250.2514 | 16780.4758 | 16598.5029 | 16625.0735 | 49 | 0.730 | 0.873 | 0.965 | 0.860 |
| 344 | -8214.1738 | 16731.1754 | 16534.3476 | 16563.0872 | 53 | 0.780 | 0.936 | 0.917 | 0.772 |
| 434 | -8219.9644 | 16742.7567 | 16545.9289 | 16574.6685 | 53 | 0.780 | 0.873 | 0.965 | 0.772 |
| 443 | -8175.4010 | 16653.6299 | 16456.8021 | 16485.5418 | 53 | 0.730 | 0.873 | 0.917 | 0.860 |
| 444 | -8145.1140 | 16615.9108 | 16404.2281 | 16435.1368 | 57 | 0.780 | 0.873 | 0.917 | 0.772 |

a: Combinations of class size of PGD-, PTSD-, and depressive-symptom trajectories, for example, Model 334 indicates 3 PGD-symptom trajectories, 3 PTSD-symptom trajectories, and 4 depressive-symptom trajectories.
b: AIC, BIC, and SABIC are penalized log-likelihood model information criteria used to compare the fit of competing models to the same data. These criteria are designed to penalize models with more parameters. AIC considers the number of parameters. BIC and SABIC account for the sample size and number of parameters.
Abbreviations: AIC: Akaike information criterion; BIC: Bayesian information criterion; SABIC: Adjusted BIC; PGD: prolonged grief disorder; PTSD: posttraumatic stress disorder. Bold indicates highest entropy for the three grief-related distress symptoms individually and as a whole.

© 2023 Wen F et al. *JAMA Network Open.*

**eFigure 1. Model fit figures for class solutions with increasing number of PGD-, PTSD-, and depressive-symptom trajectories**

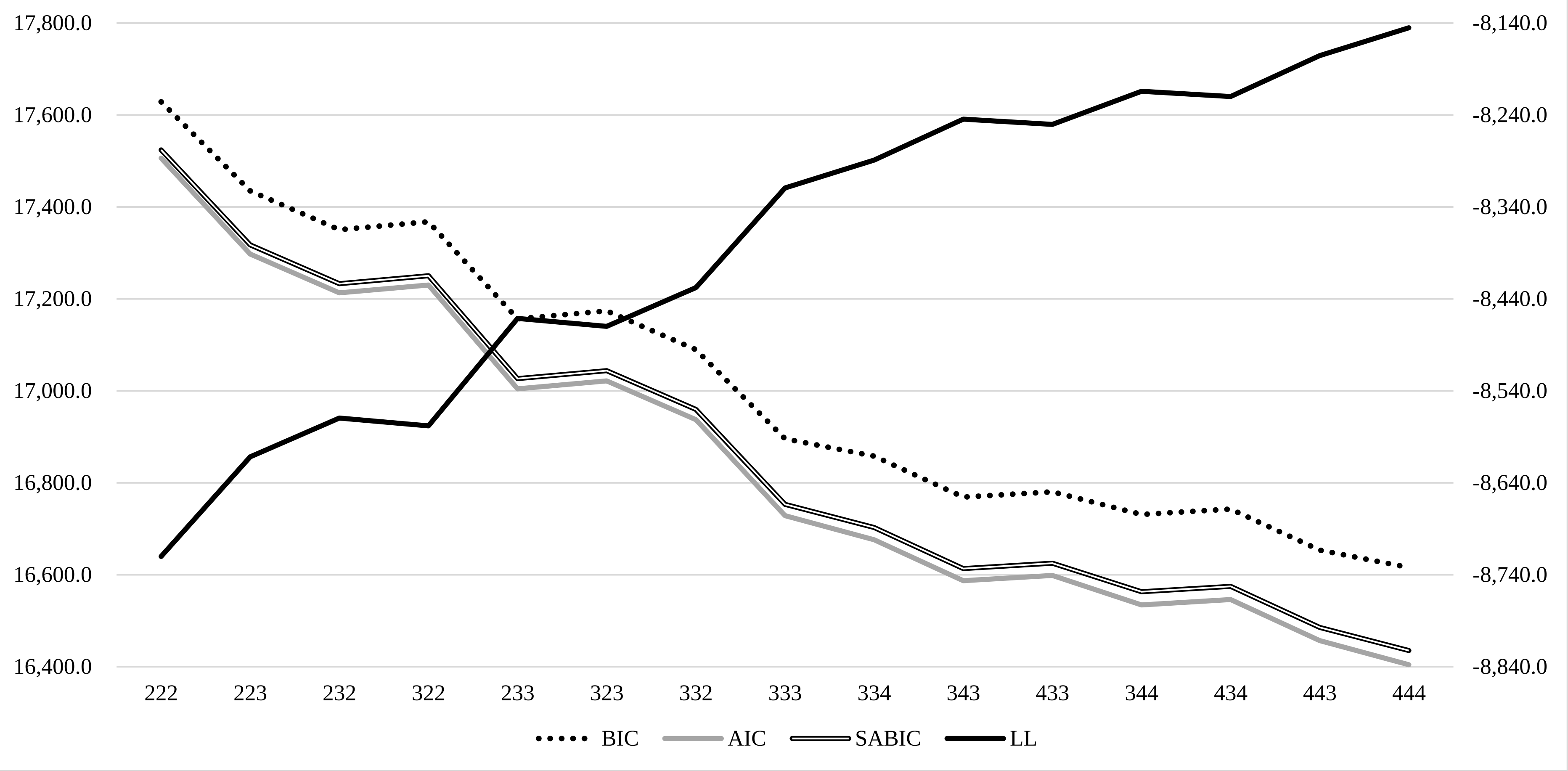


Abbreviations: PGD: Prolonged grief disorder; PTSD: posttraumatic stress disorder; BIC: Bayesian information criterion; AIC: Akaike information criterion; SABIC: sample adjusted Bayesian information criterion; LL: log-likelihood model information criterion

© 2023 Wen F et al. *JAMA Network Open.*

**eFigure 2. Co-occurrence of PGD-, PTSD-, and depressive-symptom trajectories**

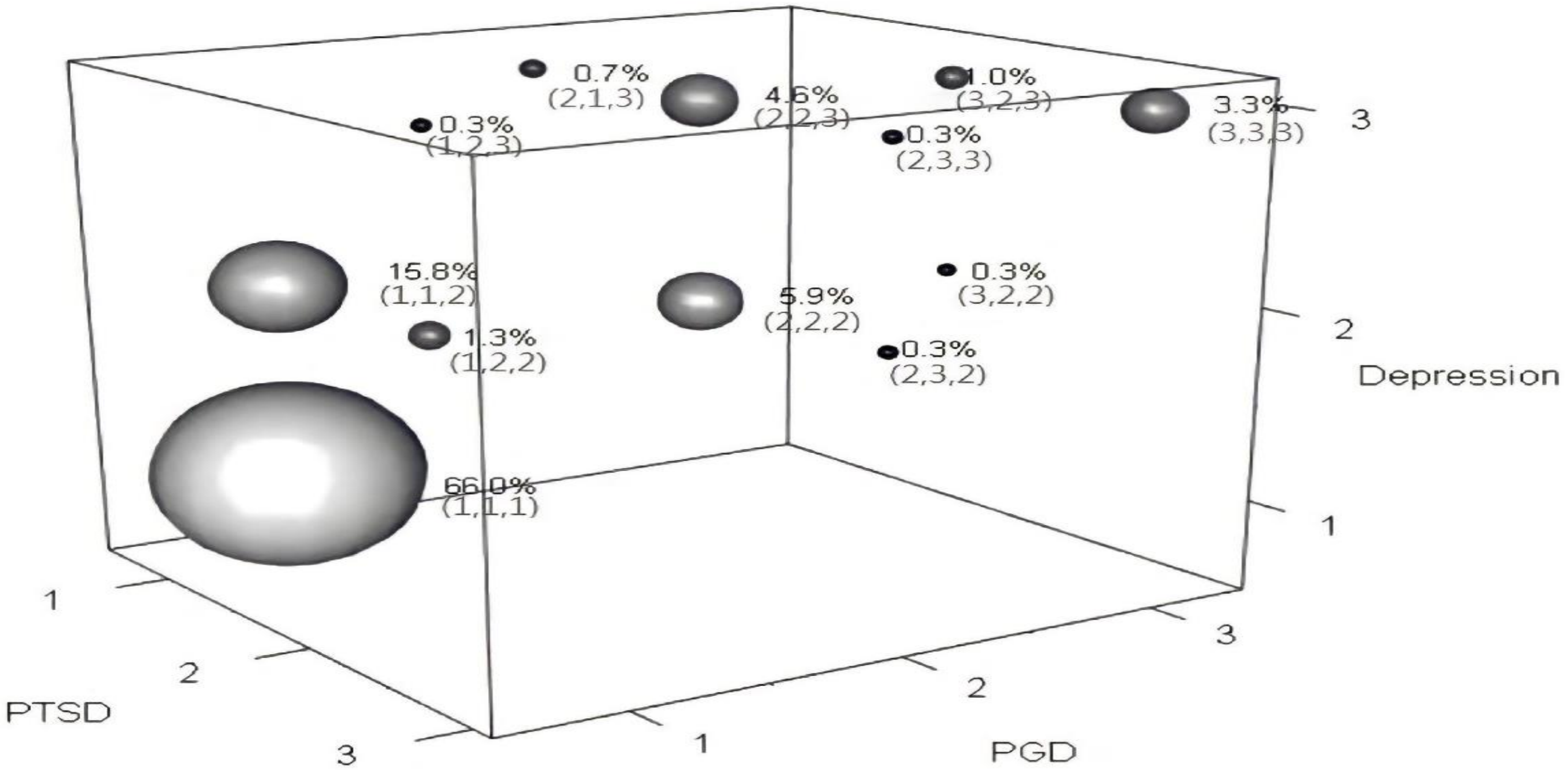


Co-occurrence (prevalence) of PGD-, PTSD-, and depressive-symptom trajectories. For example, point (1,1,2) (15.8%) indicates PGD-symptom trajectory 1, PTSD-symptom trajectory 1, and depressive-symptom trajectory 2. PGD-symptom trajectories: 1=resilience, 2=recovery, 3=chronic. PTSD-symptom trajectories: 1=resilience, 2=recovery, 3=delayed-onset. Depressive-symptom trajectories: 1=resilience, 2=moderate, 3=chronic.

© 2023 Wen F et al. *JAMA Network Open.*

**eTable 2. Model fit indices for different joint-pattern solutions of PGD-, PTSD-, and depressive-symptom trajectories**

| Model | Log-likelihood | BIC[a] | AIC[a] | SABIC[a] | # parameter | Entropy | Individual entropy | | |
|---|---|---|---|---|---|---|---|---|---|
| | | | | | | | PGD | PTSD | depression |
| 1 class | -8319.3 | 16895.7 | 16728.6 | 16753.0 | 45 | 0.615 | 0.971 | 0.991 | 0.903 |
| 2 class | -8149.8 | **16596.7** | 16403.6 | 16431.8 | 52 | 0.730 | 0.971 | 0.991 | 0.860 |
| 3 class | -8131.9 | 16601.0 | **16381.9** | **16413.9** | 59 | 0.615 | 0.971 | 0.965 | 0.903 |
| 4 class | -8131.5 | 16640.0 | 16394.9 | 16430.7 | 66 | 0.615 | 0.936 | 0.991 | 0.903 |
| 5 class | -8131.4 | 16679.8 | 16408.7 | 16448.3 | 73 | 0.730 | 0.971 | 0.965 | 0.860 |
| 6 class | -8131.3 | 16719.7 | 16422.6 | 16466.0 | 80 | 0.730 | 0.933 | 0.993 | 0.858 |

a: AIC, BIC, and SABIC are penalized log-likelihood model information criteria used to compare the fit of competing models to the same data. These criteria are designed to penalize models with more parameters. AIC considers the number of parameters. BIC and SABIC account for the sample size and number of parameters.

Bold indicates lowest values.

Abbreviations: PGD: Prolonged grief disorder; PTSD: posttraumatic stress disorder; BIC: Bayesian information criterion; AIC: Akaike information criterion; SABIC: sample adjusted Bayesian information criterion; LL: log-likelihood model information criterion.

© 2023 Wen F et al. *JAMA Network Open.*

**eFigure 3. Model fit figures for different joint-pattern solutions of PGD-, PTSD-, and depressive-symptom trajectories**

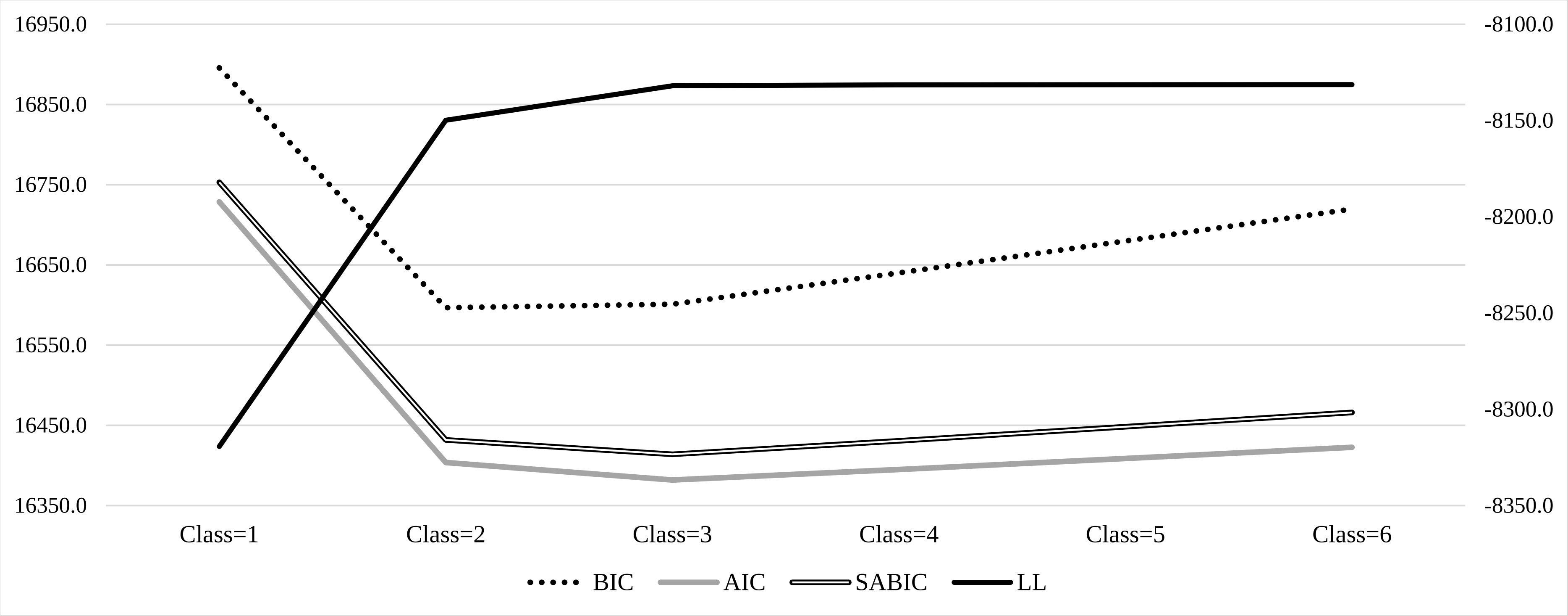


Abbreviations: PGD: Prolonged grief disorder; PTSD: posttraumatic stress disorder; BIC: Bayesian information criterion; AIC: Akaike information criterion; SABIC: sample adjusted Bayesian information criterion; LL: log-likelihood model information criterion

© 2023 Wen F et al. *JAMA Network Open.*

**eTable 3. Comparisons of identified PGD-, PTSD- and depressive-symptom trajectories across the two existing and the current studies**

| Items Study | Lenferink et al. (2020)[33] | | Djelantik et al. (2022)[34] | | Current study | |
|---|---|---|---|---|---|---|
| Sample | 172 disaster-bereaved people | | 259 bereaved people who primarily lost their loved one due to a natural cause of death | | 303 bereaved family surrogates of ICU decedents | |
| Assessment time frames | 11, 22, 31, and 42 months postdisaster | | Every six weeks from one month to 27 months post-bereavement | | 6, 13, 18, and 24 months post loss | |
| PGD-symptom trajectories (%) | mild | 81.8% | resilient | 66.4% | resilience | 83.5% |
| | | | acute recovery | 25.1% | recovery | 11.9% |
| | chronic | 18.2% | chronic | 8.4% | chronic | 4.6% |
| PTSD-symptom trajectories (%) | mild | 85.2% | resilient | 95% | resilience | 82.5% |
| | recovered | 4.2% | | | recovery | 13.5% |
| | chronic | 10.3% | chronic | 5% | | |
| | | | | | delayed-onset | 4.0% |
| Depressive-symptom trajectories (%) | mild | 85.6% | resilient | 68% | resilience | 66.0% |
| | recovered | 8.2% | | | moderate | 23.8% |
| | chronic | 6.2% | chronic | 32% | chronic | 10.2% |

© 2023 Wen F et al. *JAMA Network Open.*
