# Supplementary material for: Comorbid Prolonged Grief, PTSD, and Depression Trajectories for Bereaved Family Surrogates
Source: JAMA Netw Open. 2023 Nov 10;6(11):e2342675. doi: 10.1001/jamanetworkopen.2023.42675 (PMC10638651; doi:10.1001/jamanetworkopen.2023.42675)
Supplement: Supplement 2. — Data Sharing Statement [file jamanetwopen-e2342675-s002.pdf]

Data Sharing Statement

Wen. Comorbid Prolonged Grief, PTSD, and Depression Trajectories for Bereaved Family Surrogates. *JAMA Netw Open*. Published November 10, 2023.
doi:10.1001/jamanetworkopen.2023.42675

Data
Data available: No

Additional Information
Explanation for why data not available: The sharing of anonymized data from this study is restricted due to ethical and legal constrictions. Data contains sensitive personal health information, which is protected under The Personal Data Protection Act in Taiwan, thus making all data requests subject to Institutional Review Board (IRB) approval. Per Chang Gung Memorial Hospital (CGMH) IRB, the data that support the findings of this study are restricted for transmission to those in the primary investigative team. Data sharing with investigators outside the team requires IRB approval. All requests for anonymized data will be reviewed by the research team and then submitted to the CGMH IRB for approval.
